# Burden of informal care in stroke survivors and its determinants: a prospective observational study in an Asian setting

**DOI:** 10.1186/s12889-021-11991-3

**Published:** 2021-10-26

**Authors:** Yi Wang, Shilpa Tyagi, Helen Hoenig, Kim En Lee, Narayanaswamy Venketasubramanian, Edward Menon, Deidre Anne De Silva, Philip Yap, Boon Yeow Tan, Sherry H. Young, Yee Sien Ng, Tian Ming Tu, Yan Hoon Ang, Keng He Kong, Rajinder Singh, Reshma A. Merchant, Hui Meng Chang, Chou Ning, Angela Cheong, Gerald Choon-Huat Koh

**Affiliations:** 1 Saw Swee Hock School of Public Health, National University of Singapore, 12 Science Drive 2, #10-01, Singapore, 117549 Singapore; 2 Physical Medicine and Rehabilitation Service, Durham VA Medical Centre, 508 Fulton St, Durham, NC 27705 USA; 3 Lee Kim En Neurology Pte Ltd, Mount Elizabeth, #11-14/15, Mount Elizabeth Medical Centre, Singapore, 228510 Singapore; 4 Raffles Neuroscience Centre, Raffles Hospital, 585 North Bridge Rd, Level 9 Raffles Specialist Centre, Singapore, 188770 Singapore; 5 St. Andrew’s Community Hospital, 8 Simei Street 3, Singapore, 529895 Singapore; 6 National Neuroscience Institute, Singapore General Hospital campus, 11 Jln Tan Tock Seng, Level 1, Singapore, 308433 Singapore; 7 Dept of Geriatric Medicine, Khoo Teck Puat Hospital, 90 Central Yishun, Singapore, 768828 Singapore; 8 St. Luke’s Hospital, 2 Street 11 Bukit Batok, Singapore, 659674 Singapore; 9 Department of Rehabilitation Medicine, Changi General Hospital, 2 Simei Street 3, Singapore, 529889 Singapore; 10 Department of Rehabilitation Medicine, Singapore General Hospital, Outram Rd, Singapore, 169608 Singapore; 11 Department of Neurology, National Neuroscience Institute, Neurology, Tan Tock Seng Hospital, 11 Jln Tan Tock Seng, Level 1, Singapore, 308433 Singapore; 12 Department of Rehabilitation Medicine, Tan Tock Seng Hospital, 11 Jln Tan Tock Seng, Singapore, 308433 Singapore; 13 Department of Medicine, Yong Loo Lin School of Medicine, National University of Singapore, 10 Medical Dr, Singapore, 117597 Singapore; 14 Department of Neurosurgery, National University Hospital, 5 Lower Kent Ridge Rd, Singapore, 119074 Singapore

**Keywords:** Informal care, Quality of life, Rehabilitation, Stroke, Stroke management, Socio-economic factors

## Abstract

**Background:**

Informal caregiving is an integral part of post-stroke recovery with strenuous caregiving demands often resulting in caregiving burden, threatening sustainability of caregiving and potentially impacting stroke survivor’s outcomes. Our study aimed to examine and quantify objective and subjective informal care burden after stroke; and to explore the factors associated with informal care burden in Singapore.

**Methods:**

Stroke patients and their informal caregivers were recruited from all five tertiary hospitals in Singapore from December 2010 to September 2013. Informal care comprised of assistance provided by informal caregivers with any of the activities of daily living. Informal care burden was measured by patients’ likelihood of requiring informal care, hours of informal care required, and informal caregivers’ Zarit’s Burden Score. We examined informal care burden at 3-months and 12-months post-stroke. Generalized linear regressions were applied with control variables including patients’ and informal caregivers’ demographic characteristics, arrangement of informal care, and patients’ health status including stroke severity (measured using National Institute of Health Stroke Scale), functional status (measured using Modified Rankin Scale), self-reported depression, and common comorbidities.

**Results:**

Three hundred and five patients and 263 patients were examined at 3-months and 12-months. Around 35% were female and 60% were Chinese. Sixty three percent and 49% of the patients required informal care at 3-months and 12-months point, respectively. Among those who required informal care, average hours required per week were 64.3 h at 3-months and 76.6 h at 12-months point. Patients with higher functional dependency were more likely to require informal care at both time points, and required more hours of informal care at 3-months point. Female informal caregivers and those caring for patients with higher functional dependency reported higher Zarit’s Burden. While informal caregivers who worked full-time reported higher burden, those caring for married stroke patients reported lower burden at 3-months point. Informal caregivers who co-cared with foreign domestic workers, i.e.: stay-in migrant female waged domestic workers, reported lower burden.

**Conclusions:**

Informal care burden remains high up to 12-months post-stroke. Factors such as functional dependency, stroke severity, informal caregiver gender and co-caring with foreign domestic workers were associated with informal care burden.

**Supplementary Information:**

The online version contains supplementary material available at 10.1186/s12889-021-11991-3.

## Background

Globally, stroke ranked third in causing years of life lost in 2017 [1]. The high magnitude of economic burden of stroke has been reported in many countries [2–6]. In Singapore, stroke is one of the leading contributors to the burden of disease [7]. Direct medical cost due to stroke imposes considerable economic burden in Singapore [8].

Informal caregivers, the role usually played by family members, close relatives or friends, are often required to assist patients at home with their post-stroke care needs. Significant amount of time can be required from informal caregivers [9, 10]. A study in Spain found that average daily hours required for informal care were 8.7 h 3 months post stroke and 7.2 h 12 months post stroke [10]. From the societal perspective, this can impose considerable economic strain [11–13]. A study based in UK reported that the cost of one stroke to NHS increased to £29,405 from £15,306 over 5 years when informal caregiving costs were incorporated [14]. Another estimate from the US suggested the national burden of informal caregiving associated with injurious falls in stroke survivors to be $2.9 billion [15]. The total national economic burden associated with informal caregiving post-stroke was reported to be $14.2 billion in another US based study [16].

At the same time, informal caregivers may experience stress, strain and decreased quality of life [17–20]. Factors affecting informal care burden have been explored in the literature with mixed results. Association between higher functional dependency of the patients and the higher informal caregivers’ burden was found in some studies [9, 10, 21] but not in others [22]. More hours of care provided [10, 18], higher number of tasks [17], and worse patients’ mental health [9, 19] were found to be associated with higher informal caregivers’ strain. Patients’ and informal caregivers’ demographic characteristics were also reported to be associated with the burden in some studies [23, 24]. Family support and patients’ factors [18, 19] were found to be associated with informal caregivers’ burden at some specific time points after stroke but not the other time points.

Though the literature is abundant, no such study has been conducted in Singapore examining the informal care burden post-stroke and its potential determinants. In Singapore, patients with stroke symptoms usually seek care at the emergency department of any of the public tertiary hospitals after which they are admitted for appropriate treatment. Once stabilized in acute care setting, they can either be discharged to a step-down setting (locally known as community hospitals) or discharged home. The average length of stay after stroke in such public tertiary hospitals is reported to be about 7.7 days [25]. While in acute care setting, stroke survivors are also assessed for eligibility for rehabilitation and based on this assessment they may undergo intensive rehabilitation in an inpatient setting. Alternatively, they may be referred to supervised community rehabilitation (either after inpatient rehabilitation or as an alternative to the same), often delivered at the day rehabilitation centres spread across whole of Singapore. It has been reported previously that more than 70% of the stroke survivors are discharged home from acute care setting [26], highlighting the relevance of informal care provided at home. Different from Western settings, foreign domestic workers (FDWs) can be employed in Singapore to help care for patients with stroke. A FDW is “a stay-in migrant female waged domestic worker attached to one employer and works for only a single household, under Singapore’s strict legal permit system” [27]. They are usually hired to provide assistance in daily housework. When households’ members get sick, the FDWs can play important roles in informal caregiving [28].

From a societal perspective, quantifying informal care burden is important to estimate the total cost of stroke. Moreover, highlighting the determinants of informal care burden along with its magnitude can contribute to designing policies and social support interventions to help patients and their informal caregivers post-stroke. To address the aforementioned gaps, this study aimed to examine burden of informal care of patients with stroke and its potential determinants at 3-months point and 12-months point post-stroke in Singapore. Specific objectives of the study are as follows: (1) to quantify the hours of informal caregiving reported at 3-months point and 12-months point post stroke, (2) to examine the determinants of receiving informal care, (3) to examine the determinants of amount of informal care received and (4) to examine the determinants of informal caregiver burden reported at 3-months point and 12-months point post stroke.

## Methods

### Data sources

Singapore Stroke Study (S3) was a yearlong prospective study in Singapore with the recruitment period from December 2010 to September 2013. Stroke patients and their informal caregivers were recruited from all five tertiary hospitals in Singapore. Stroke patients were eligible if they were: (i) Singaporean or permanent resident, more than 40 years old and residing in Singapore for the next 1 year, (ii) stroke must be a recent diagnosis (i.e. stroke symptoms occurring within 4 weeks prior to admission) with diagnosis made by a clinician and/or supported by brain imaging (CT or MRI) and (iii) not globally aphasic. An informal caregiver could be an immediate or extended family member or friend, more than 21 years (the legal definition of adult in Singapore), providing care or assistance of any kind and taking responsibility for the patient, as recognized by the patient and not fully paid for caregiving. We defined the informal caregivers that participated S3 as corresponding informal caregivers. FDWs were not eligible to be corresponding informal caregivers. S3 was approved by the National University of Singapore Institutional Review Board, SingHealth Centralized Institutional Review Board and the National Health Group Domain Specific Review Board. Written informed consent was obtained from both the patients and informal caregivers. Further details of S3 are reported elsewhere [29].

### Data collection

Patients and corresponding informal caregivers were interviewed face-to-face at baseline, 3-months point, and 12-months point post stroke. Corresponding informal caregivers were also interviewed by phone at 6-months point and 9-months point. Information collected included patients’ and corresponding informal caregivers’ demographics, healthcare resource utilization, health-related information for both patients and corresponding informal caregivers, and time spent caring for stroke patients by the assisting informal caregivers defined as the informal caregivers that provided informal care in assisting stroke patients with their daily activities. A corresponding informal caregiver may or may not be an assisting informal caregiver. For example, the corresponding informal caregiver could provide other types of assistance such as financial assistance or monitoring the FDWs to assist patients with their daily activities. Overtime, some of the patients no longer required informal care. The corresponding informal caregivers were still followed up to collect data.

Standardised questionnaires and forms, piloted using 40 participants, were used to collect data. The interviews were conducted by a team of research assistants, who underwent a 3-days training to learn about the content and right method of administering the survey and interview. To maximise response rates, efforts were made to accommodate participants’ schedules and reminders were sent out before each interview. Multiple attempts (up to three) were made to reach out to participants.

This work used data collected at 3-months point and 12-months point, when information from both patients and corresponding informal caregivers were available.

### Dependent variables

The dependent variables included hours of informal care received by the patients and the Zarit’s Burden Score [30] of corresponding informal caregivers. While informal care was defined as *“*providing care or assistance of any kind and taking responsibility for the patient, as recognized by the patient and not fully paid for caregiving*”* when recruiting corresponding informal caregivers*,* in this work, we focused on examining burden due to informal care comprising of assistance provided by the assisting informal caregivers with any of the activities of daily living (i.e.: doing light housework, preparing meals, eating, bathing or showering, dressing, using the toilet, getting in or out of a bed or chair, walking, using private or public transport, shopping, using the telephone, taking medicine as prescribed, and minor healthcare activities which do not require medical training etc.) in the past 3 months due to any physical or mental health problem experienced by the stroke survivor. For each patient, the corresponding informal caregiver reported the average hours of informal care per week provided by all the assisting informal caregivers during the past 3 months at 3-months point and 12-months point. For each assisting informal caregiver, we capped the hours of care provided per week at 84 h. We added up the average weekly hours provided by all the assisting informal caregivers to generate the total number of “person-hours” of care received by each patient per week.

Corresponding informal caregiver’s appraisal of caregiving burden was captured by the Zarit’s Burden Interview, which involved asking corresponding informal caregivers to rate how often they felt several negatively phrased questions related to their caregiving role [31]. Validated previously in Singapore [32], we used the abbreviated 12-item version for the current study with a total score ranging from 0 to 48. Higher score implied higher burden. Different cut-offs, such as 13, 17, 18, have been proposed in the literature to detect caregivers with high or severe burden [33–35]. To avoid making arbitrary decision in cut-off point and avoid loss of information associated with dividing continuous variables into categories, we kept Zarit’s Burden Score as continuous variable in this study*.*

Zarit’s Burden Interview was one part of the survey questions for the corresponding informal caregivers in S3. The corresponding informal caregivers can choose not to answer the Zarit’s Burden Interview if they did not provide any informal care. In this work, we focused on the burden due to assistance with stroke patients’ daily activities. Zarit’s Burden Score were only examined for the corresponding informal caregivers who were assisting informal caregivers and assisted stroke patients with their daily activities.

### Independent variables

Guided by the literature, independent variables selected included patients’ and corresponding caregivers’ demographic characteristics [23, 24]; patients’ level of dependency, severity of stroke, and other comorbidities [9, 10, 21]; patients’ mental health [9]; hours of informal care provided by the assisting informal caregivers and co-caring with other assisting informal caregivers [18, 20]. Co-caring with FWDs was also included to fit Singapore’s context. Comorbidities collected in S3 included cardiovascular disease, diabetes, hypertension, hyperlipidemia, chronic lung disease, dementia, autoimmune disease, leukaemia, liver disease, malignant solid tumor, and renal disease. Common comorbidities including cardiovascular disease, diabetes, hypertension, and hyperlipidemia were included in the analysis. Patients were categorized as either having or not having the corresponding comorbidities. The remaining comorbidities were not included in the analysis due to very low prevalence rate among the S3 patients.

For patients, demographic information including age, gender, ethnicity, and marital status were used. Ward class recorded at recruitment was used as a proxy for socio-economic status of the patients. There are four types of ward classes in public hospitals in Singapore, A, B1, B2 and C, with increasing level of subsides. The quality of care across 4 classes is similar, but the level of amenities differs. For example, ward class C has up to 8 beds in a room. Ward class A is a single room with attached bathroom and toilet, television, fully automated electric bed, choice of meal, and sleeper unit for accompanying adult at additional charge. The daily rate for different wards are around 35 SGD (~ 26 USD) for ward class C, 79 SGD (~ 59 USD) for ward class B2, 251 SGD (~ 186 USD) for ward class B1, and 466 SGD (~ 346 USD) for ward class A. Patients and/or patients’ representatives can choose the ward class to stay. Patients living in ward class B1 and A were expected to have higher social economic status compared to patients living in ward class B2 and ward class C. Modified Rankin Scale (MRS) [36] measured at 3-months point and 12-months point were used to measure the level of disability and dependence of the patients. The score was classified into 3 categories in this study: no symptoms or no significant disability despite symptoms (0–1), slight disability or moderate disability (2–3), and moderately severe disability or severe disability (4–5). National Institute of Health Stroke Scale (NIHSS) [37] and other comorbidities including cardiovascular disease, diabetes, hypertension, and hyperlipidaemia collected at recruitment were used. NIHSS was classified into 2 categories in this study: moderate to severe stroke with score equal and higher than 5, and mild with score less than 5. Information of whether the patients’ self-reporting depressive symptoms at 3-months point and 12-months point were also used.

For corresponding informal caregivers who were interviewed, their gender, age, ethnicity, and marital status were collected. Corresponding informal caregivers reported their relationship with the patients and their working status, as well as of the other assisting informal caregivers who provided care to the patients.

### Statistical analysis

While descriptive statistics of person-hours of informal care provided were presented for all the assisting informal caregivers, Zarit’s Burden Score was only summarized and examined using regression analysis for the corresponding informal caregivers who were assisting informal caregivers at the same time. Multivariable regression analysis was conducted to examine the factors associated with informal care burden at 3-months point and 12-months point. Logistic regression was used to examine the requirement of informal care. Generalized linear regression was used to examine the hours of informal care required and the Zarit’s Burden Score. In all the analyses, a full model including all the relevant control variables was used first. A parsimonious model was then generated by iteratively removing the most insignificant control variables until all the variables were with *p* < 0.10. However, we maintained patients’ demographic information, informal caregivers’ demographic information, and patients’ MRS in the parsimonious specification regardless of the *p*-value. We reported the results from parsimonious models in the main text. The results from full models can be found from Supplemental Material Table III and Table IV. All analyses were conducted using Stata Statistical Software: Release 16 [38].

## Results

**Fig. 1:**
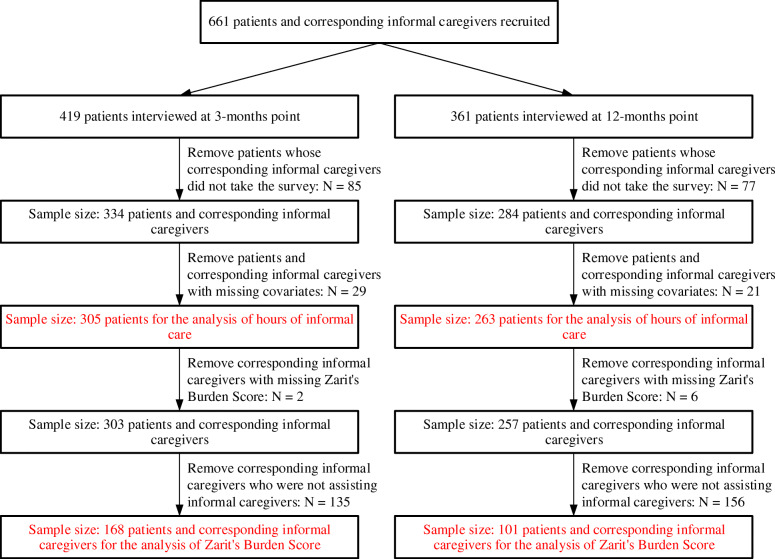
Study Sample Flowchart

At the baseline, 661 patients with stroke were recruited. After removing patients and corresponding informal caregivers with missing information, 305 (46%) and 263 (40%) patients were used to analyse the hours of care at 3-months point and 12-months point, respectively. For the Zarit’s Burden Score, the number of corresponding informal caregivers, who were assisting informal caregivers at the same time, were 168 at 3-months point and 101 at 12-months point. We used the term assisting informal caregivers when discussing the results on Zarit’s Burden Score to highlight the burden was driven by informal care in assisting stroke patients with their daily activities. The study flowchart was presented in Fig. 1.

Table 1 shows the demographic information of patients and corresponding informal caregivers included in the analysis for hours of care. Distribution of patients’ gender, age, ethnicity, marital status, ward class, were similar at 3-months points and 12-months points based on statistical tests. Majority of the stroke patients were male, 66.6% at 3-months point and 65.0% at 12-months point. Fifty nine point 7 % of the patients were Chinese at 3-months point and 63.1% of patients were Chinese at 12-months point. Majority of the patients were married. At 3-months point, 27.5% of the patients were with slight or moderate disability; and 18.4% of the patients were with moderate severe or severe disability. At 12-months point, 22.1% of the patients were with slight or moderate disability; and 16.7% of the patients were with moderate severe or severe disability. Available information at recruitment was also presented to understand the participants without follow-ups or with missing information. Characteristics, that were significantly different between the participants that were followed up and not followed up, were highlighted. Detailed comparison was presented in Supplemental Material Table I.

**Table 1:** Summary Statistics of Demographic Information

	3-months point post-stroke, Number (%)	12-months point post-stroke, Number (%)	At recruitment, Number (%)
Patients			
Number	305	263	661
Gender			
Male	203 (66.6%)	171 (65.0%)	437 (66.1%)
Female	102 (33.4%)	92 (35.0%)	224 (33.9%)
Age			
< = 50	35 (11.7%)	33 (12.7%)	90 (13.6%)
51–65	150 (47.0%)	133 (48.9%)	321 (48.6%)
> 65	120 (41.3%)	97 (38.4%)	250 (37.8%)
Ethnicity			
Chinese	182 (59.7%)*	166 (63.1%)	446 (67.5%)
Malay, Indian, and others	123 (40.3%)*	97 (36.9%)	215 (32.5%)
Married	239 (78.4%)*	204 (77.6%)*	452 (68.4%)
Ward class			
(Unsubsidized) A & B1	20 (6.6%)	17 (6.4%)	48 (7.3%)
(Subsidized) B2	138 (45.2%)	123 (46.8%)	285 (43.1%)
(Subsidized) C	147 (48.2%)	123 (46.8%)	328 (49.6%)
Modified Rankin Scale			
No symptoms or No significant disability despite symptoms (0–1)	165 (54.1%)	161 (61.2%)	NA
Slight disability or Moderate disability (2–3)	84 (27.5%)	58 (22.1%)	NA
Moderate severe disability or severe disability (4–5)	56 (18.4%)	44 (16.7%)	NA
Depressed	96 (31.5%)	56 (21.3%)	NA
First-time stroke	248 (81.3%)	213 (81.0%)	533 (80.8%)
NIHSS score at recruitment			
No stroke symptom or minor stroke (0–4)	165 (54.1%)*	146 (55.5%)	373 (60.0%)
Moderate, moderate severe, or sever stroke (5–42)	140 (45.9%)*	117 (44.5%)	249 (40.0%)
Other comorbidities at recruitment			
Cardiovascular disease	60 (19.7%)	45 (17.1%)	121 (18.3%)
Diabetes	133 (43.6%)	118 (44.9%)	274 (41.5%)
Hypertension	220 (72.1%)	197 (74.9%)	480 (72.6%)
Hyperlipidemia	222 (72.8%)	185 (70.3%)	473 (71.6%)
Corresponding Informal Caregivers			
Number	305	263	NA
Gender			
Male	77 (25.3%)	66 (25.1%)	NA
Female	228 (74.7%)	197 (74.9%)	NA
Age			
< = 50	137 (44.9%)	115 (43.7%)	NA
51–65	126 (41.3%)	105 (39.9%)	NA
> 65	42 (13.8%)	43 (16.4%)	NA
Ethnicity			
Chinese	181 (59.3%)	164 (62.4%)	NA
Malay, Indian, and others	124 (40.7%)	99 (37.6%)	NA
Married	241 (79.0%)	212 (81.5%)	NA
Relationship with Patients			
Spouse	178 (58.4%)	126 (64.0%)	NA
Children, children-in-law, grandchildren	99 (32.5%)	55 (27.9%)	NA
Parents	5 (1.6%)	2 (1.0%)	NA
Other relatives and friends	23 (7.5%)	14 (7.1%)	NA
Living in the same house with the patients	274 (89.9%)	227 (86.1%)	NA

Notes: * The characteristic are significantly different at 5% between the participants that were followed-up and not followed-up. Detailed comparison can be found in Supplemental Material Table I

Table 2 shows the summary statistics about informal care burden. Among all the patients, 62.6% required informal care over the last 3 months as reported at 3-months interview and 48.7% required informal care over the last 3 months as reported at 12-months interview. Among the patients that required informal care, the average hours per week required were 64.3 h at 3-months point and 76.6 h at 12-months point. The median hours per week required were 56 h at 3-months point and 60 h at 12-months point. The total number of informal caregivers involved in caring for the patients during the past 3 months were 324 at 3-months point and 246 at 12-months point. Additional information on the hours of informal care provided per informal caregiver and the histogram of hours of informal care provided per week were presented in Supplemental Material Table II and Fig. I. The total hours of informal care per week could be greater than the total number of hours per week for the patients with more than 1 assisting informal caregiver.

**Table 2:** Summary Statistics for Informal Care Burden in the Past 3 Months

	3-months point post-stroke, Number (%)	12-months point post-stroke, Number (%)
Number of patients	305	263
Number of patients required informal care	191 (62.6%)	128 (48.7%)
Hours required per week per patient for those who required informal care		
Mean (SD)	64.3 (52.9) hours	76.6 (78.2) hours
Median (5th percentile, 95th percentile)	56 (7, 182) hours	60 (7, 189) hours
Number of assisting informal caregivers per patient for those who required informal care		
1	99 (51.8%)	65 (50.8%)
2	58 (30.4%)	28 (21.9%)
3 and above	34 (17.8%)	35 (27.3%)
FDWs as assisting informal caregivers		
1 assisting informal caregiver:		
Non-FDW	90 (47.1%)	59 (46.1%)
FDW	9 (4.7%)	6 (4.7%)
More than 1 assisting informal caregiver		
Without FDW	55 (28.8%)	51 (39.8%)
With FDW	37 (19.4%)	12 (9.4%)
Zarit’s Burden Score of assisting informal caregivers		
Number of corresponding informal caregivers who were assisting informal caregivers	168	101
Mean (SD)	9.2 (7.6)	8.6 (7.9)
Median (5th percentile, 95th percentile)	8 (0, 22)	7 (0, 22)

Among the patients who required informal care, 168 out of their 191 (88.0%) corresponding informal caregivers and 101 out of their 128 (78.9%) corresponding informal caregivers were assisting informal caregivers at 3-months point and 12-months point respectively. The mean and median for Zarit’s Burden Score were 9.2 and 8 at 3-months point. At 12-months point, the mean and median for Zarit’s Burden Score were 8.6 and 7. Assisting informal caregivers reported higher burden at 3-months point compared to 12-months point. Histogram of Zarit’s Burden Score was presented in Supplemental Material Fig. II.

Table 3 shows results examining the factors associated with requiring informal care and hours of informal care required. Higher MRS was associated with higher likelihood of requiring informal care at both 3-months point and 12-months point. At 3-months point, compared with patients with no symptom or disability, the odds ratio (OR) of requiring informal care was 2.69 (95%-CI = (1.43, 5.06)) and 6.33 (95%-CI = (2.44, 16.40)) for patients with slight or moderate disability and moderate severe or severe disability, respectively. At 12-months point, compared with patients with no symptom or disability, the OR of requiring informal care was 3.58 (95%-CI = (1.84, 6.94)) and 10.63 (95%-CI = (4.33, 26.05)) for patients with slight or moderate disability and moderate severe or severe disability, respectively.

**Table 3:** Regression: Factors associated with Requirement of Informal Care and Hours of Informal Care – Parsimonious Model

	3-Months Point		12-Months Point	
	Requirement of Informal Care	Hours of Informal care	Requirement of Informal Care	Hours of Informal care
Number of Observations	305	191	263	128
	OR (95%-CI)	Coefficient (95%-CI)	OR (95%-CI)	Coefficient (95%-CI)
Age				
Age group: <= 50	–	–	–	–
Age group: 51–65	0.96 (0.42, 2.18)	−0.18 (− 0.60, 0.23)	1.58 (0.65, 3.83)	0.49 (− 0.10, 1.08)
Age group: > 65	1.61 (0.67, 3.85)	− 0.16 (− 0.60, 0.29)	1.81 (0.71, 4.61)	0.53 (− 0.08, 1.14)*
Ethnicity: Chinese	1.40 (0.82, 2.39)	0.07 (− 0.18, 0.33)	0.88 (0.49, 1.59)	0.07 (− 0.29, 0.42)
Gender: Female	1.41 (0.78, 2.54)	0.27 (0.001, 0.55)**	1.22 (0.66, 2.25)	0.02 (−0.32, 0.37)
Married	1.48 (0.73, 3.00)	−0.07 (− 0.38, 0.24)	0.81 (0.40, 1.64)	0.07 (− 0.33, 0.47)
Ward class				
Ward class: A&B1	1.86 (0.57, 6.12)	0.07 (−0.41, 0.54)	2.97 (0.83, 10.60)*	0.10 (−0.48, 0.67)
Ward class: B2	1.00 (0.58, 1.73)	−0.01 (− 0.27, 0.24)	0.74 (0.41, 1.33)	0.38 (0.02, 0.74)**
Ward class: C	–	–	–	–
Modified Rankin Scale				
Modified Rankin Scale: No symptom or disability	–	–	–	–
Modified Rankin Scale: slight or moderate disability	2.69 (1.43, 5.06)***	0.30 (0.02, 0.58)**	3.58 (1.84,6.94)***	−0.35 (−0.77, 0.07)
Modified Rankin Scale: moderate severe or severe disability	6.33 (2.44, 16.40)***	0.54 (0.23, 0.86)***	10.63 (4.33, 26.05)***	−0.02 (− 0.43, 0.40)
NIHSS: moderate to severe stroke	1.75 (0.98, 3.13)*	0.35 (0.09, 0.60)***	NA	NA
Hyperlipidemia	NA	NA	1.76 (0.94, 3.28)*	NA
Depressed	1.73 (0.97, 3.11)*	NA	NA	NA

Notes: * Significant at 0.10; ** significant at 0.05; *** significant at 0.01

“NA” means the independent variable was not included in the parsimonious model. The independent variables were omitted in the table if they were included in none of the parsimonious model. Please refer to the Supplemental Material for the results from the full model

“- “indicates the reference category

At 3-months point, compared to patients with no symptom or disability, patients with slight or moderate disability and patients with moderate severe or severe disability required more hours of informal care per week. Patients with moderate to severe stroke also required more hours of informal care per week (Coef = 0.35, 95%-CI = (0.09, 0.60)). Compared to male patients, female patients required more hours of informal care (Coef = 0.27, 95%-CI = (0.001, 0.65)) at 3-months point. At 12-months point, most of the factors were not associated with the hours required for informal care. Compared to patients in ward class C, patients from ward class B2 required more hours of informal care (Coef = 0.38, 95%-CI = (0.02, 0.74)).

Table 4 shows the results of the factors associated with Zarit’s Burden Score. Using parsimonious model, at 3-months point, the scores were higher (Coef = 0.60, 95%-CI = (0.29, 0.91)) for assisting informal caregivers taking care of patients with moderate severe or severe disability compared to assisting informal caregivers taking care of patients with no symptoms or disability. Assisting informal caregivers taking care of depressed patients reported higher score (Coef = 0.34, 95%-CI = (0.09, 0.58)). Assisting informal caregivers reported lower scores (Coef = − 0.34, 95%-CI = (− 0.66, − 0.02)) if the patients were married. Female assisting informal caregivers reported higher score (Coef = 0.39, 95%-CI = (0.04, 0.75)) as compared to male assisting informal caregivers. Compared to part-time workers, unemployed people, and retired people, full-time workers reported higher score (Coef = 0.29, 95%-CI = (0.05, 0.54)). If there were FDWs co-caring for the patients, the assisting informal caregivers reported lower score (Coef = − 0.62, 95%-CI = (− 0.97, − 0.28)). Surprisingly, hours of care provided was not associated with Zarit’s Burden Score. Also, co-caring with people other than the FDWs had no impact on Zarit’s Burden Score.

**Table 4:** Regression: Factors associated with Zarit’s Burden Score – Parsimonious Model

	3-Months Point	12-Months Point
Number of Observations	168	101
	Coefficient (95%-CI)	Coefficient (95%-CI)
Covariates: Patients		
Patient’s Age		
Patients’ age group: <= 50	–	–
Patients’ age group: 51–65	0.25 (−0.15, 0.66)	− 0.28 (−1.05, 0.48)
Patients’ age group: > 65	0.24 (− 0.18, 0.66)	−0.32 (−1.06, 0.42)
Patients’ ethnicity: Chinese	− 0.05 (− 0.29, 0.19)	−0.03 (− 0.41, 0.35)
Patients’ gender: Female	0.05 (− 0.27, 0.37)	−0.43 (− 0.87, 0.02)*
Patients’: married	−0.34 (− 0.66, − 0.02)**	0.27 (− 0.2, 0.74)
Ward class		
Ward class: A&B1	−0.47 (− 0.99, 0.06)*	−0.02 (− 0.72, 0.69)
Ward class: B2	− 0.07 (− 0.34, 0.19)	0.13 (− 0.31, 0.56)
Ward class: C	–	–
Modified Rankin Scale		
Modified Rankin Scale: No symptom or disability	–	–
Modified Rankin Scale: slight or moderate disability	0.27 (−0.002, 0.53)*	0.47 (0.002, 0.94)**
Modified Rankin Scale: moderate severe or severe disability	0.60 (0.29, 0.91)***	0.75 (0.29, 1.21)***
Hypertension	NA	0.69 (0.23, 1.16)***
Depressed	0.34 (0.09, 0.58)***	NA
Covariates: Caregivers		
Caregivers’ Age		
Caregivers’ age group: <= 50	–	–
Caregivers’ age group: 51–65	0.05 (−0.23, 0.32)	0.35 (−0.11, 0.81)
Caregivers’ age group: > 65	− 0.28 (− 0.64, 0.09)	0.33 (− 0.21, 0.87)
Caregivers’ gender: Female	0.39 (0.04, 0.75)**	0.61 (0.09, 1.14)**
Caregivers’ work status: full-time workers	0.29 (0.05, 0.54)**	NA
Co-care with foreign domestic workers	−0.62 (− 0.97, − 0.28)***	NA

Notes: * Significant at 0.10; ** significant at 0.05; *** significant at 0.01

Caregivers in this table means the corresponding informal caregivers who were assisting informal caregivers at the same time

“NA” means the independent variable was not included in the parsimonious model. The independent variables were omitted in the table if they were included in none of the parsimonious model. Please refer to the Supplemental Material for the results from the full model

“- “indicates the reference category

At 12-months point, compared to assisting informal caregivers caring for patients with no symptoms or disability, assisting informal caregivers caring for patients with slight or moderate disability and moderate severe to severe disability reported higher scores. Assisting informal caregivers caring for patients having hypertension at recruitment also reported higher scores (Coef = 0.69, 95%-CI = (0.23, 1.16)). Female assisting informal caregivers reported higher scores (Coef = 0.61, 95%-CI = (0.09, 1.14)).

## Discussion

With the aim to examine the informal care burden post-stroke and its determinants at 3-months point and 12-months point, we presented a comprehensive account of informal care burden for post-stroke patients in Singapore. Overall, the number of stroke patients requiring informal care decreased over 1-year period post stroke. But for those who required informal care, they required more hours of informal care on average at 12-months point.

A reasonably large proportion of stroke patients had multiple informal caregivers within the Asian context of Singapore where family caregiving is prevalent. The role played by family members in providing care to their loved ones is in line with the Singaporean principles of families being the “first line of support” with the community and government stepping in where necessary. In Southeast Asia, family is perceived as a unit comprising a web of familial social networks, centered around elderly parents and adult children residing in the same or different households; care is a functional activity (work, task) with a relational process (love, thinking, doing). Familial care (care within the family boundary) may extend beyond immediate family members [39].

At 12-months point, level of disability and dependency was found to be associated with likelihood of patients requiring informal care only, but not the hours of informal care. At 12-months point, the assisting informal caregivers could be familiar with the caring routine and become more efficient in caring the patients. The assisting informal caregivers may enter adaption phase as time passes in which they become confident in their ability to support activities of daily living [40]. The hours of informal care depends on the demand of the stroke patients as well as the efficiency level of the assisting informal caregivers. The functional dependency of the patients could have small impact on the hours of informal care by the time of 12-months point.

We reported both stroke survivor and assisting informal caregiver characteristics as significant determinants of assisting informal caregivers’ perceived burden of care. Functional status as measured on the MRS was significantly associated with the Zarit’s Burden Score at both 3-months point and 12-months point. In concordance with past literature [41–43], we found higher level of functional disability to be associated with higher Zarit’s Burden Score as compared to stroke survivors with lower level of functional disability. It is possible that the caregiving responsibilities may increase in tandem with the functional limitations post-stroke making caregiving endeavour more intense and demanding. Moreover, it may be emotionally stressful to witness one’s family member going through the challenging post-stroke recovery trajectory. Together these may influence the assisting informal caregiver’s appraisal of their caregiving role. Stroke survivor’s depressive symptoms were significantly associated with the Zarit’s Burden Score at 3-months point. This is consistent with existing literature reporting significant association between stroke survivor’s and caregiver’s mental health [9, 19]. We found presence of hypertension in patients was associated with informal caregivers reporting higher caregiving burden. Possible explanation could be related to additional care needs associated with having a comorbid condition like hypertension. Significant association between patients’ increasing level of comorbidity and informal caregiver burden has been reported in patients with cognitive impairment [44].

The informal caregiver characteristics found significant in current analysis seem to reflect the unique caregiving landscape of Singapore, with caregiving responsibilities shared among FDWs and family informal caregivers. Co-care with FDWs was associated with lower Zarit’s Burden Score at 3-months point only and not at 12-months point. It is well-known that first 3 months post-stroke are more challenging for the caregivers as they familiarize themselves with the new caregiving responsibilities and the uncertain care needs of the stroke survivors [29, 45]. Thus, assistance from FDWs may play a relatively more crucial role in the early post-stroke period as compared to late post-stroke period. Similar to our finding. Chong and colleagues [46] reported FDWs as a moderator of distress in caregivers of frail elders. While co-care with FDWs was significantly associated with lower Zarit’s Burden Score, co-care with other assisting informal caregivers was not significantly associated with Zarit’s Burden Score. Possible explanations could be related to the broad scope of duties performed by the FDWs within their employer’s home, ranging from providing childcare, eldercare to engaging in other household chores [47]. Thus, FDWs may help with competing commitments of the assisting informal caregiver, resulting in reduced overall strain on the assisting informal caregiver. This facilitation role of the FDWs, also described as “*an important coping resource for caregivers*” and contributing to “*lessen the intensity of stressors*” in literature [48], may or may not be replicated in co-care by family members, as family members may primarily want to be engaged in caregiving responsibilities. The significance of our finding is further highlighted by the fact that caregivers receiving assistance from FDWs during first 3 months is also associated with improved stroke survivor outcomes [25].

We found married stroke survivors had assisting informal caregivers reporting lower caregiver burden at 3-months point as compared to their unmarried counterparts. The protective effect of marital status on health outcomes of both stroke [49] and non-stroke population [50] has been reported in past literature. Moreover, within the stroke population, Liu and colleagues [51] recently reported married stroke survivors having favourable outcomes post-stroke as compared to those unmarried. It may be possible that married stroke survivors with lower stroke related disability and recurrence may have relatively lower care needs resulting in lower perceived burden by their caregivers.

Female assisting informal caregivers reported higher Zarit’s Burden Score at both 3-months point and 12-months point. Similar results have been reported in the literature [52, 53]. Female assisting informal caregivers may take on more challenging and tedious caregiving duties resulting in higher caregiver burden as compared to their male counterparts. At 3-months point, full-time workers reported higher score compared to part-time workers, unemployed and retired. Full-time workers may experience Double-burden effect due to competing commitments required from work and caring for stroke patients [54, 55].

Adopting a comprehensive approach, we conceptualized burden of informal care for current study to include both objective (“number of hours of care”) and subjective (“Zarit’s Burden Score”) components. This not only enabled us to report the significant determinants of both objective and subjective burden of informal care, but also explore the relationship between the two. Interestingly, we found no significant association between hours of care and Zarit’s Burden Score, which is contrary to the findings of some researchers [10, 18, 20]. One possibility could be related to the differences between objective and subjective caregiving burden estimates. The latter comprises of caregiver’s appraisal of the caregiving role, which often incorporates other parameters like role limitations due to mental or physical health of caregivers [56], sense of coherence [57], competing commitments of the caregivers, etc. In fact, in a non-stroke population, the association between objective and subjective caregiver burden was reported to be “*complex*”, with subjective caregiver burden encompassing not just the amount of caregiving but also the caregiver-care recipient relationship dynamics [58]. Also, it is important to note that hours of caregiving solely may not be indicative of the caregiving burden, and the nature of caregiving denoted by the constituting caregiving tasks [41] may be more strongly associated with perceived caregiving burden. While we did not capture caregiving hours spent on specific caregiving tasks in current study, we recommend future research efforts capture both hours of caregiving and specific caregiving activities to further expand on our findings. The burden due to different activities could differ, for example comparing bathing and shower to taking medicine. In the scenario of multiple informal caregivers being available, overall burden of informal care could be reduced by optimal allocation of types of assistance. Although we found that “number of hours of care” was not associated with “Zarit’s Burden Score”, it is important to acknowledge that both are important for policy making. “Number of hours of care” has financing and economic implications while informal caregiver burden is a strong determinant of caregiver burnout, caregiver abuse and premature admission to nursing home which itself has financing and economic implications.

Using a yearlong cohort consisting of stroke patients and their informal caregivers in Singapore, our study contributed to the existing literature of burden of informal care due to stroke considering an Asian setting. Our study documented some unique results including the prevalence of multiple informal caregivers and FDWs. We also observed results that were different from the literature, e.g.: hours of informal care was not associated with informal caregivers’ subjective burden. Further study could be conducted to understand the reasons. While generalizability of findings related to co-care with FDWs may be limited to similar settings as Singapore (where FDWs are a common occurrence), most of the other findings of our study have relatively greater generalizability. Findings related to association between functional status post-stroke and caregiver burden, gender of informal caregiver and marital status of stroke survivors are generalizable to other settings.

Our study has several limitations. First, corresponding informal caregivers reported the hours of informal care provided by all the assisting informal caregivers. But, efforts were made to minimize the recall bias and collect high quality data including training the data collectors, using standardised questionnaire and forms, and setting the recall period as 3 months. Second, patients self-reported whether they were depressed. Third, though efforts were made to follow up the participants in S3, not all the recruited patients who were alive at 3-months point and 12-months point were followed up. Overall mortality rate was less than 5%, which is unlikely to introduce a selection bias [29]. For the patients included in the analysis, most of the demographic characteristics and clinical characteristics were similar at recruitment, at 3-months point, and at 12-months point as shown in Table 1. Married patients were more likely to be followed up, which could be due to the fact that spouse plays an important role in informal care. Chinese patients were less likely to be followed up. Future studies can investigate this issue. Furthermore, compared to the patients at recruitment, smaller proportion of patients, lower by around 5%, were with NIHSS indicating “No stroke symptom or minor stroke” at 3-months and 12-months point. One potential explanation is that patients who were lost to follow up were those who did not require informal care or required low level of informal care. In such a case, patients with low NIHSS and who required low level of informal care were more likely lost to follow-up and omitted from our sample, which can lead to overestimation of overall informal care burden and underestimation of the association between NIHSS and informal care burden. Fourth, we focused on examining the factors associated with the likelihood of patients requiring informal care, hours of informal care required and the care burden of informal caregivers broadly. We did not explore the detailed explanations and causal pathways behind. Further work should be conducted to better understand this. However, our work can serve as a road map to guide such work. Fifth, the care burden of FDWs were not examined, as FDWs were not eligible to be corresponding informal caregivers. Future studies can address this gap.

## Conclusions

Our study shows informal care burden remains high up to 12-months point post-stroke. Both patient factors and informal caregiver factors were associated with likelihood of requiring informal care, hours of informal care required, and subjective caregivers’ burden. Our results provide empirical evidence for policymakers to consider when designing polices to support patients and informal caregivers in post-stroke caregiving in the community.

## Supplementary Information

**Additional file 1.**

## Data Availability

The anonymized data that support the findings of this study are available from the corresponding author upon reasonable request.

## Abbreviations

CI: confidence interval
Coef: coefficient
FDW: foreign domestic worker
MRS: Modified Rankin Scale
NIHSS: National Institute of Health Stroke Scale
OR: odds ratio
S3: Singapore Stroke Study
