# Supplementary material for: Burden of informal care in stroke survivors and its determinants: a prospective observational study in an Asian setting
Source: BMC Public Health. 2021 Oct 26;21:1945. doi: 10.1186/s12889-021-11991-3 (PMC8547090; doi:10.1186/s12889-021-11991-3)
Supplement: Supplementary file 1 — Additional file 1. [file 12889_2021_11991_MOESM1_ESM.docx]

**Supplemental Material**

**Burden of Informal Care in Stroke Survivors and its Determinants: A Prospective Observational Study in an Asian Setting**

**Authors and Affiliations:**

Yi Wang, PhD

Saw Swee Hock School of Public Health, National University of Singapore

12 Science Drive 2, #10-01, Singapore 117549, Singapore

Shilpa Tyagi, PhD

Saw Swee Hock School of Public Health, National University of Singapore

12 Science Drive 2, #10-01, Singapore 117549, Singapore

Helen Hoenig, MD

Physical Medicine and Rehabilitation Service, Durham VA Medical Centre, USA

508 Fulton St, Durham, NC 27705, United States

Kim En Lee, FRCP

Lee Kim En Neurology Pte Ltd, Singapore

3 Mount Elizabeth, #11-14/15, Mount Elizabeth Medical Centre, Singapore 228510

Narayanaswamy Venketasubramanian, FRCP

Raffles Neuroscience Centre, Raffles Hospital, Singapore

585 North Bridge Rd, Level 9 Raffles Specialist Centre, Singapore 188770

Edward Menon, FAMS

St. Andrew’s Community Hospital, Singapore

8 Simei Street 3, Singapore 529895

Deidre Anne De Silva, FRCP

National Neuroscience Institute, Singapore General Hospital campus, Singapore

11 Jln Tan Tock Seng, Level 1, Singapore 308433

Philip Yap, MRCP

Dept of Geriatric Medicine, Khoo Teck Puat Hospital, Singapore

90 Yishun Central, Singapore 768828

Boon Yeow Tan, FCFP

St. Luke's Hospital, Singapore

2 Bukit Batok Street 11, Singapore 659674

Sherry H. Young, M.D.

Department of Rehabilitation Medicine, Changi General Hospital, Singapore

2 Simei Street 3, Singapore 529889

Yee Sien Ng, MRCP

Department of Rehabilitation Medicine, Singapore General Hospital, Singapore

Outram Rd, Singapore 169608

Tian Ming Tu, MRCP

Department of Neurology, National Neuroscience Institute, Neurology, Tan Tock Seng Hospital, Singapore

11 Jln Tan Tock Seng, Level 1, Singapore 308433

Yan Hoon Ang, FRCP

Geriatric Medicine, Khoo Teck Puat Hospital, Singapore

90 Yishun Central, Singapore 768828

Keng He Kong, MRCP

Department of Rehabilitation Medicine, Tan Tock Seng Hospital, Singapore

11 Jln Tan Tock Seng, Singapore 308433

Rajinder Singh, FRCP

Department of Neurology, National Neuroscience Institute, Neurology, Tan Tock Seng Hospital, Singapore

11 Jln Tan Tock Seng, Level 1, Singapore 308433

Reshma A. Merchant, FRCP

Department of Medicine, Yong Loo Lin School of Medicine, National University of Singapore

10 Medical Dr, Singapore 117597

Hui Meng Chang, FAMS

National Neuroscience Institute, Singapore General Hospital campus, Singapore

11 Jln Tan Tock Seng, Level 1, Singapore 308433

Chou Ning, FRCS

Department of Neurosurgery, National University Hospital, Singapore

5 Lower Kent Ridge Rd, Singapore 119074

Angela Cheong, BSN

Saw Swee Hock School of Public Health, National University of Singapore, Singapore

12 Science Drive 2, #10-01, Singapore 117549, Singapore

Gerald Choon-Huat Koh, PhD

Ministry of Health Office for Healthcare Transformation & Saw Swee Hock School of Public Health, National University of Singapore

12 Science Drive 2, #10-01, Singapore 117549, Singapore

**Corresponding author**:

Gerald Choon-Huat Koh, PhD

Saw Swee Hock School of Public Health, National University of Singapore, 12 Science Drive 2, #10-01, Singapore 117549, Singapore

Email: ephkohch@nus.edu.sg

**Table I**: Comparison of Demographic Information Between Participants Followed-up and not Followed-up Amongst All the Participants Recruited at Baseline

|  | 3-months point post-stroke,  Number (%) | | | 12-months point post-stroke, Number (%) | | |
| --- | --- | --- | --- | --- | --- | --- |
|  | Included in the study | Not included in the study | P-value | Included in the study | Not included in the study | P-value |
| Number | 305 | 356 |  | 263 | 398 |  |
| Gender |  |  |  |  |  |  |
| Male | 203 (66.6%) | 234 (65.7%) | 0.823 | 171 (65.0%) | 266 (66.8%) | 0.629 |
| Female | 102 (33.4%) | 122 (34.3%) |  | 92 (35.0%) | 132 (33.2%) |  |
| Age |  |  |  |  |  |  |
| <= 50 | 35 (11.7%) | 55 (15.5%) | 0.317 | 33 (12.7%) | 57 (14.3%) | 0.660 |
| 51 - 65 | 150 (47.0%) | 171 (48.0%) |  | 133 (48.9%) | 188 (47.2%) |  |
| > 65 | 120 (41.3%) | 130 (36.5%) |  | 97 (38.4%) | 153 (38.4%) |  |
| Ethnicity |  |  |  |  |  |  |
| Chinese | 182 (59.7%) | 264 (74.2%) | <0.001 | 166 (63.1%) | 280 (70.4%) | 0.052 |
| Malay, Indian, and others | 123 (40.3%) | 92 (25.8%) |  | 97 (36.9%) | 118 (29.6%) |  |
| Married | 239 (78.4%) | 213 (59.8%) | <0.001 | 204 (77.6%) | 248 (62.3%) | <0.001 |
| Ward class |  |  |  |  |  |  |
| (Unsubsidized) A & B1 | 20 (6.6%) | 28 (7.9%) | 0.545 | 17 (6.4%) | 31 (7.8%) | 0.294 |
| (Subsidized) B2 | 138 (45.2%) | 147 (41.3%) |  | 123 (46.8%) | 162 (40.7%) |  |
| (Subsidized) C | 147 (48.2%) | 181(50.8%) |  | 123 (46.8%) | 205 (51.5%) |  |
| First-time stroke | 248 (81.3%) | 285 (80.3%) | 0.738 | 213 (81.0%) | 320 (80.6%) | 0.902 |
| NIHSS score at recruitment |  |  |  |  |  |  |
| No stroke symptom or minor stroke (0 - 4) | 165 (54.1%) | 208 (65.6%) | 0.003 | 146 (55.5%) | 227 (63.2%) | 0.052 |
| Moderate, moderate severe, or sever stroke (5 - 42) | 140 (45.9%) | 109 (34.4%) |  | 117 (44.5%) | 132 (36.8%) |  |
| Other comorbidities at recruitment |  |  |  |  |  |  |
| Cardiovascular disease | 60 (19.7%) | 61 (17.1%) | 0.400 | 45 (17.1%) | 76 (19.1%) | 0.518 |
| Diabetes | 133 (43.6%) | 141 (39.6%) | 0.298 | 118 (44.9%) | 156 (39.2%) | 0.147 |
| Hypertension | 220 (72.1%) | 260 (73.0%) | 0.795 | 197 (74.9%) | 283 (71.1%) | 0.284 |
| Hyperlipidemia | 222 (72.8%) | 251 (70.5%) | 0.517 | 185 (70.3%) | 288 (72.4%) | 0.573 |

**Table II**: Additional Summary Statistics of All the Informal Caregivers

|  | 3-months point post-stroke | 12-months point post-stroke |
| --- | --- | --- |
| Number of informal caregivers | 324 | 246 |
| Relationship with Patients |  |  |
| Spouse | 119 (36.7%) | 67 (27.2%) |
| Children, children-in-law, grandchildren | 130 (40.1%) | 129 (52.4%) |
| Parents | 4 (1.2%) | 6 (2.4%) |
| Other relatives and friends | 23 (7.1%) | 26 (10.6%) |
| Foreign domestic workers | 48 (14.8%) | 18 (7.3%) |
| Working status |  |  |
| Full-time workers | 114 (35.2%) | 108 (43.9%) |
| Part-time workers | 34 (10.5%) | 34 (13.8%) |
| Unemployed or retired | 128 (39.5%) | 86 (35.0%) |
| Foreign domestic workers | 48 (14.8%) | 18 (7.3%) |
| Hours taking care of patients per week per informal caregiver | Mean (Median) | Mean (Median) |
| All | 36.3 (24.5) hours | 37.4 (30) hours |
| Relationship with Patients |  |  |
| Spouse | 39.1 (25) hours | 45.9 (42) hours |
| Children, children-in-law, grandchildren | 26.0 (15) hours | 26.9 (24) hours |
| Parents | 66.5 (84) hours | 60.7 (67) hours |
| Other relatives and friends | 37.7 (21) hours | 40.6 (33) hours |
| Foreign domestic workers | 53.7 (58) hours | 68.0 (84) hours |
| Working status (excluding foreign domestic workers) |  |  |
| Full-time workers | 27.2 (15) hours | 28.1 (24) hours |
| Part-time workers | 32.0 (22.5) hours | 38.3 (38) hours |
| Unemployed or retired | 38.9 (28) hours | 42.2 (42) hours |

**Table III**: Regression: Factors associated with Requirement of Informal Care and Hours of Informal Care – Full Model

|  | 3-Months Point | | 12-Months Point | |
| --- | --- | --- | --- | --- |
|  | Requirement of Informal Care | Hours of Informal care | Requirement of Informal Care | Hours of Informal care |
| Number of Observations | 305 | 191 | 263 | 128 |
|  | OR (95%-CI) | Coefficient (95%-CI) | OR (95%-CI) | Coefficient (95%-CI) |
| Age |  |  |  |  |
| Age group: <= 50 | - | - | - | - |
| Age group: 51 - 65 | 0.96 (0.42, 2.21) | -0.19 (-0.62, 0.23) | 1.46 (0.59, 3.60) | 0.46 (-0.15, 1.08) |
| Age group: > 65 | 1.55 (0.63, 3.78) | -0.15 (-0.60, 0.29) | 1.73 (0.67, 4.48) | 0.52 (-0.12, 1.17) |
| Ethnicity: Chinese | 1.36 (0.79, 2.37) | 0.04 (-0.22, 0.31) | 0.87 (0.47, 1.59) | 0.11 (-0.26, 0.49) |
| Gender: Female | 1.45 (0.79, 2.67) | 0.29 (0.02, 0.57)** | 1.25 (0.66, 2.37) | 0.06 (-0.33, 0.44) |
| Married | 1.46 (0.71, 2.99) | -0.06 (-0.38, 0.25) | 0.84 (0.41, 1.72) | 0.07 (-0.36, 0.50) |
| Ward class |  |  |  |  |
| Ward class: A&B1 | 1.70 (0.52, 5.58) | 0.14 (-0.36, 0.63) | 2.79 (0.77, 10.11) | 0.04 (-0.55, 0.63) |
| Ward class: B2 | 0.93 (0.53, 1.63) | 0.004 (-0.26, 0.27) | 0.77 (0.42, 1.40) | 0.31 (-0.09, 0.70) |
| Ward class: C | - | - | - | - |
| Modified Rankin Scale |  |  |  |  |
| Modified Rankin Scale: No symptom or disability | - | - | - | - |
| Modified Rankin Scale: slight or moderate disability | 2.98 (1.55, 5.72)*** | 0.28 (-0.02, 0.57)* | 3.24 (1.52, 6.90)*** | -0.31 (-0.78, 0.17) |
| Modified Rankin Scale: moderate severe or severe disability | 7.45 (2.75, 20.20)*** | 0.56 (0.23, 0.90)*** | 8.66 (3.13, 23.93)*** | -0.02 (-0.49, 0.45) |
| First-time stroke | 1.50 (0.72, 3.09) | -0.01 (-0.33, 0.31) | 0.95 (0.45, 2.02) | 0.05 (-0.40, 0.50) |
| NIHSS: moderate to severe stroke | 1.74 (0.96, 3.13)* | 0.32 (0.05, 0.59)** | 1.32 (0.72, 2.40) | 0.18 (-0.19, 0.56) |
| Cardiovascular disease | 0.70 (0.35, 1.38) | 0.07 (-0.23, 0.37) | 1.29 (0.59, 2.83) | -0.01 (-0.44, 0.42) |
| Diabetes | 0.87 (0.49, 1.54) | -0.14 (-0.40, 0.13) | 1.08 (0.60, 1.94) | 0.10 (-0.26, 0.47) |
| Hypertension | 1.10 (0.58, 2.07) | 0.002 (-0.31, 0.32) | 0.69 (0.33, 1.43) | -0.12 (-0.62, 0.37) |
| Hyperlipidemia | 1.46 (0.75, 2.85) | 0.07 (-0.25, 0.38) | 1.92 (0.92, 3.98) | 0.25 (-0.23, 0.74) |
| Depressed | 1.67 (0.93, 3.02)* | 0.15 (-0.11, 0.41) | 1.09 (0.50, 2.39) | -0.22 (-0.65, 0.21) |

Notes: * Significant at 0.10; ** significant at 0.05; *** significant at 0.01.

**Table IV**: Regression: Factors associated with Zarit’s Burden Score – Full Model

|  | 3-Months Point | 12-Months Point |
| --- | --- | --- |
| Number of Observations | 168 | 101 |
|  | Coefficient (95%-CI) | Coefficient (95%-CI) |
| Covariates: Patients | | |
| Patient’s Age |  |  |
| Patients' age group: <= 50 | - | - |
| Patients' age group: 51 - 65 | 0.24 (-0.20, 0.68) | -0.26 (-1.09, 0.58) |
| Patients' age group: > 65 | 0.26 (-0.20, 0.71) | -0.29 (-1.13, 0.55) |
| Patients' ethnicity: Chinese | -0.02 (-0.28, 0.24) | 0.03 (-0.41, 0.47) |
| Patients' gender: Female | 0.01 (-0.33, 0.36) | -0.50 (-1.02, 0.03)* |
| Patients': married | -0.34 (-0.68, -0.003)** | 0.22 (-0.30, 0.74) |
| Ward class |  |  |
| Ward class: A&B1 | -0.54 (-1.13, 0.05)* | -0.06 (-0.91, 0.79) |
| Ward class: B2 | -0.10 (-0.39, 0.18) | 0.11 (-0.41, 0.62) |
| Ward class: C | - | - |
| Modified Rankin Scale |  |  |
| Modified Rankin Scale: No symptom or disability | - | - |
| Modified Rankin Scale: slight or moderate disability | 0.17 (-0.16, 0.49) | 0.59 (0.01, 1.17)** |
| Modified Rankin Scale: moderate severe or severe disability | 0.45 (0.06, 0.84)** | 0.68 (0.04, 1.32)** |
| First-time stroke | 0.02 (-0.32, 0.37) | 0.13 (-0.41, 0.68) |
| NIHSS: moderate to severe stroke | 0.16 (-0.11, 0.43) | 0.31 (-0.14, 0.76) |
| Cardiovascular disease | -0.01 (-0.34, 0.32) | -0.27 (-0.84, 0.30) |
| Diabetes | 0.10 (-0.15, 0.36) | 0.02 (-0.45, 0.49) |
| Hypertension | 0.20 (-0.11, 0.51) | 0.60 (0.06, 1.15)** |
| Hyperlipidemia | -0.08 (-0.41, 0.26) | 0.24 (-0.32, 0.80) |
| Depressed | 0.32 (0.06, 0.59)** | 0.31 (-0.21, 0.83) |
| Covariates: Caregivers | | |
| Caregivers' Age |  |  |
| Caregivers' age group: <= 50 | - | - |
| Caregivers' age group: 51 - 65 | 0.06 (-0.24, 0.35) | 0.36 (-0.15, 0.87) |
| Caregivers' age group: > 65 | -0.27 (-0.66, 0.12) | 0.34 (-0.28, 0.96) |
| Caregivers' gender: Female | 0.42 (0.03, 0.80)** | 0.50 (-0.17, 1.17) |
| Caregivers’ work status: full-time workers | 0.30 (0.03, 0.58)** | -0.06 (-0.60, 0.49) |
| Hours of care provided |  |  |
| Hours of care provided: <=28 hours/week | -0.11 (-0.46, 0.24) | 0.04 (-0.53, 0.61) |
| Hours of care provided: 28 - 56 hours/week | 0.12 (-0.18, 0.42) | 0.24 (-0.35, 0.82) |
| Hours of care provided: > 56 hours/week | - | - |
| Co-caring Status |  |  |
| Co-care with others | 0.05 (-0.25, 0.35) | 0.28 (-0.23, 0.78) |
| Co-care with foreign domestic workers | -0.60 (-1.01, -0.19)*** | -0.35 (-1.15, 0.45) |

Notes: * Significant at 0.10; ** significant at 0.05; *** significant at 0.01

Caregivers in this table means the corresponding informal caregivers who were assisting informal caregivers at the same time.

**Table V**: Regression: Factors associated with Hours of Informal Care - Marginal Effect

|  | 3-Months Point | | 12-Months Point | |
| --- | --- | --- | --- | --- |
|  | Full model | Parsimonious model | Full model | Parsimonious model |
| Number of Observations | 191 | 191 | 128 | 128 |
|  | Marginal effect (95%-CI) | Marginal effect (95%-CI) | Marginal effect (95%-CI) | Marginal effect (95%-CI) |
| Age |  |  |  |  |
| Age group: <= 50 | - | - | - | - |
| Age group: 51 - 65 | -13.5 (-44.7, 17.8) | -12.7 (-43.5, 18.0) | 28.6 (-4.8, 62.0)* | 30.4 (-1.3, 62.1)* |
| Age group: > 65 | -10.8 (-43.7, 22.0) | -10.8 (-43.4, 21.7) | 33.2 (-2.8, 69.3)* | 33.2 (-0.5, 66.9)* |
| Ethnicity: Chinese | 2.8 (-14.2, 19.8) | 4.8 (-11.2, 20.8) | 8.7 (-19.7, 37.1) | 5.1 (-21.7, 31.8) |
| Gender: Female | 19.5 (0.4, 38.7)** | 18.1 (-0.7, 36.8)* | 4.3 (-25.5, 34.2) | 1.7 (-24.9, 28.4) |
| Married | -4.2 (-25.3, 16.9) | -4.8 (-25.6, 16.0) | 5.1 (-27.0, 37.2) | 5.3 (-24.6, 35.2) |
| Ward class |  |  |  |  |
| Ward class: A&B1 | 9.4 (-26.3, 45.1) | 4.5 (-28.2, 37.1) | 2.7 (-37.2, 42.7) | 6.3 (-32.6, 45.1) |
| Ward class: B2 | 0.3 (-16.6, 17.1) | -0.8 (-17.2, 15.6) | 23.6 (-7.0, 54.2) | 28.8 (0.67, 56.9)** |
| Ward class: C | - | - | - | - |
| Modified Rankin Scale |  |  |  |  |
| Modified Rankin Scale: No symptom or disability | - | - | - | - |
| Modified Rankin Scale: slight or moderate disability | 15.5 (-1.2, 32.2)* | 16.9 (0.68, 33.1)** | -22.1 (-56.0, 11.9) | -24.9 (-54.7, 5.0) |
| Modified Rankin Scale: moderate severe or severe disability | 36.9 (12.8, 61.0)*** | 35.1 (12.8, 57.4)*** | -1.8 (-40.9, 37.3) | -1.4 (-36.0, 33.1) |
| First-time stroke | -0.7 (-21.4, 20.1) | NA | 4.0 (-29.5, 37.6) | NA |
| NIHSS: moderate to severe stroke | 20.0 (3.6, 36.4)** | 21.5 (5.8, 37.2)*** | 14.0 (-14.0, 41.9) | NA |
| Cardiovascular disease | 4.7 (-15.7, 25.2) | - | -0.7 (-33.7, 32.3) | - |
| Diabetes | -8.8 (-25.8, 8.1) | NA | 8.0 (-19.8, 35.8) | NA |
| Hypertension | 0.1 (-20.2, 20.5) | NA | -9.9 (-50.4, 30.6) | NA |
| Hyperlipidemia | 4.2 (-15.4, 23.8) | NA | 18.0 (-14.4, 50.4) | NA |
| Depressed | 9.8 (-7.9, 27.6) | NA | -16.2 (-46.2, 13.9) | NA |

Notes: * Significant at 0.10; ** significant at 0.05; *** significant at 0.01. This table shows the marginal effect of results in Table 3.

“NA” means the independent variable was not included in the parsimonious model. The independent variables were omitted in the table if they were included in none of the parsimonious model.

“-“ indicates the reference category.

**Table VI**: Factors associated with Zarit’s Burden Score - Marginal Effect

|  | **3-Months Point** | | **12-Months Point** | |
| --- | --- | --- | --- | --- |
|  | Full Model | Parsimonious Model | Full Model | Parsimonious Model |
| Number of Observations | 168 | 168 | 101 | 101 |
|  | Marginal effect (95%-CI) | Marginal effect (95%-CI) | Marginal effect (95%-CI) | Marginal effect (95%-CI) |
| Covariates: Patients | | | | |
| Patient’s Age |  |  |  |  |
| Patients' age group: <= 50 | - | - | - | - |
| Patients' age group: 51 - 65 | 2.0 (-1.5, 5.5) | 2.1 (-1.1, 5.3) | -2.6 (-12.0, 6.7) | -2.9 (-11.5, 5.8) |
| Patients' age group: > 65 | 2.2 (-1.4, 5.8) | 2.0 (-1.3, 5.3) | -2.9 (-12.2, 6.4) | -3.2 (-11.6, 5.2) |
| Patients' ethnicity: Chinese | -0.2 (-2.6, 2.2) | -0.5 (-2.8, 1.8) | 0.2 (-3.7, 4.2) | -0.3 (-3.7, 3.1) |
| Patients' gender: Female | 0.1 (-3.1, 3.4) | 0.5 (-2.6, 3.5) | -4.1 (-8.3, 0.1)* | -3.5 (-7.0, -0.004)** |
| Patients': married | -3.4 (-7.3, 0.4)* | -3.4 (-7.0, 0.1)* | 1.8 (-2.2, 5.9) | 2.2 (-1.4, 5.8) |
| Ward class |  |  |  |  |
| Ward class: A&B1 | -4.2 (-8.1, -0.3)** | -3.7 (-7.2, -0.1)** | -0.5 (-7.5, 6.6) | -0.2 (-6.0, 5.7) |
| Ward class: B2 | -1.0 (-3.7, 1.7) | -0.7 (-3.2, 1.9) | 1.0 (-3.7, 5.7) | 1.1 (-2.8, 5.1) |
| Ward class: C | - | - | - | - |
| Modified Rankin Scale |  |  |  |  |
| Modified Rankin Scale: No symptom or disability | - | - | - | - |
| Modified Rankin Scale: slight or moderate disability | 1.4 (-1.3, 4.1) | 2.2 (-0.1, 4.4)* | 5.0 (-0.6, 10.5)* | 3.7 (-0.5, 7.9)* |
| Modified Rankin Scale: moderate severe or severe disability | 4.4 (0.4, 8.4)** | 5.9 (2.4, 9.5)*** | 6.0 (-0.6, 12.6)* | 7.0 (1.7, 12.3)*** |
| First-time stroke | 0.2 (-2.9, 3.4) | NA | 1.2 (-3.4, 5.7) | NA |
| NIHSS: moderate to severe stroke | 1.5 (-0.9, 3.9) | NA | 2.7 (-1.4, 6.8) | NA |
| Cardiovascular disease | -0.1 (-3.1, 3.0) | NA | -2.3 (-6.8, 2.2) | NA |
| Diabetes | 1.0 (-1.4, 3.4) | NA | 0.2 (-4.0, 4.4) | NA |
| Hypertension | 1.8 (-0.9, 4.4) | NA | 4.6 (0.7, 8.6)** | 5.2 (1.9, 8.5)*** |
| Hyperlipidemia | -0.7 (-4.0, 2.5) | NA | 2.1 (-2.4, 6.6) | NA |
| Depressed | 3.0 (0.4, 5.7)** | 3.2 (0.7, 5.6)** | 2.9 (-2.5, 8.4) | NA |
| Covariates: Caregivers | | | | |
| Caregivers' Age |  |  |  |  |
| Caregivers' age group: <= 50 | - | - | - | - |
| Caregivers' age group: 51 - 65 | 0.6 (-2.3, 3.4) | 0.5 (-2.2, 3.1) | 3.2 (-1.6, 8.0) | 3.1 (-1.2, 7.3) |
| Caregivers' age group: > 65 | -2.2 (-5.3, 0.8) | -2.3 (-5.1, 0.6) | 2.9 (-3.1, 9.0) | 2.9 (-2.3, 8.0) |
| Caregivers' gender: Female | 3.4 (0.6, 6.2)** | 3.2 (0.6, 5.8)** | 3.8 (-0.8, 8.5) | 4.5 (1.0, 8.1)** |
| Caregivers’ work status: full-time workers | 2.9 (0.1, 5.8)** | 2.8 (0.3, 5.4)** | -0.5 (-5.3, 4.3) |  |
| Hours of care provided |  |  |  |  |
| Hours of care provided: <=28 hours/week | -1.0 (-3.9, 2.0) | NA | 0.4 (-4.4, 5.1) | NA |
| Hours of care provided: 28 - 56 hours/week | 1.2 (-1.8, 4.1) | NA | 2.2 (-3.3, 7.6) | NA |
| Hours of care provided: > 56 hours/week | - | NA | - | NA |
| Co-caring Status |  |  |  |  |
| Co-care with others | 0.4 (-2.4, 3.2) | NA | 2.5 (-2.2, 7.3) | NA |
| Co-care with foreign domestic workers | -4.7 (-7.6, -1.8)*** | -4.9 (-7.3, -2.4)*** | -2.7 (-8.3, 2.9) | NA |

Notes: * Significant at 0.10; ** significant at 0.05; *** significant at 0.01. This table shows the marginal effect of the results in Table 4.

Caregivers in this table means the corresponding informal caregivers who were assisting informal caregivers at the same time.

“NA” means the independent variable was not included in the parsimonious model. The independent variables were omitted in the table if they were included in none of the parsimonious model.

“-“ indicates the reference category.

**Table VII**: Full-model Using NIHSS with Three Categories

|  | **3-Months Point** | |
| --- | --- | --- |
|  | Requirement of Informal Care | Hours of Informal Care |
| Number of Observations | 305 | 191 |
|  | OR (95%-CI) | Coefficient (95%-CI) |
| Patient’s Age |  |  |
| Patients' age group: <= 50 | - | - |
| Patients' age group: 51 - 65 | 0.96 (0.42, 2.20) | -0.19 (-0.61, 0.23) |
| Patients' age group: > 65 | 1.53 (0.63, 3.75) | -0.15 (-0.60, 0.30) |
| Patients' ethnicity: Chinese | 1.37 (0.79, 2.38) | 0.40 (-0.23, 0.31) |
| Patients' gender: Female | 1.47 (0.80, 2.73) | 0.26 (-0.02, 0.54) |
| Patients': married | 1.45 (0.71, 2.98) | -0.90 (-0.41, 0.23) |
| Ward class |  |  |
| Ward class: A&B1 | 1.68 (0.51, 5.56) | 0.16 (-0.34, 0.66) |
| Ward class: B2 | 0.93 (0.53, 1.63) | 0.001 (-0.26, 0.26) |
| Ward class: C | - | - |
| Modified Rankin Scale |  |  |
| Modified Rankin Scale: No symptom or disability | - | - |
| Modified Rankin Scale: slight or moderate disability | 3.01 (1.56, 5.80)*** | 0.26 (-0.04, 0.56)* |
| Modified Rankin Scale: moderate severe or severe disability | 7.90 (2.73, 22.86)*** | 0.50 (0.15, 0.85)** |
| First-time stroke | 1.48 (0.72, 3.08) | 0.02 (-0.31, 0.34) |
| NIHSS |  |  |
| No to minor stroke | - | - |
| Moderate stroke | 1.77 (0.97, 3.21)* | 0.28 (0.01, 0.55)** |
| Moderate severe to severe stroke | 1.37 (0.31, 6.03) | 0.58 (0.09, 1.06)** |
| Cardiovascular disease | 0.70 (0.35, 1.39) | 0.07 (-0.23, 0.37) |
| Diabetes | 0.87 (0.49, 1.54) | -0.12 (-0.39, 0.14) |
| Hypertension | 1.09 (0.58, 2.06) | 0.01 (-0.30, 0.33) |
| Hyperlipidemia | 1.46 (0.75, 2.85) | 0.08 (-0.24, 0.39) |
| Depressed | 1.67 (0.93, 3.01)* | 0.14 (-0.12, 0.41) |

Notes: * Significant at 0.10; ** significant at 0.05; *** significant at 0.01.

This table shows the results categorizing NIHSS into 3 categories. Compared to categorizing NIHSS into 2 categories, different results were only obtained for hours of informal care at 3-months point.

**Table VIII**: Full Model Considering Full-time Workers, Part-time Workers, Unemployed and Retired

|  | 3-Months Point |
| --- | --- |
| Number of Observations | 168 |
|  | Coefficient (95%-CI) |
| Patient’s Age |  |
| Patients' age group: <= 50 | - |
| Patients' age group: 51 - 65 | 0.24 (-0.21, 0.69) |
| Patients' age group: > 65 | 0.26 (-0.20, 0.72) |
| Patients' ethnicity: Chinese | -0.02 (-0.28, 0.24) |
| Patients' gender: Female | 0.01 (-0.33, 0.36) |
| Patients': married | -0.34 (-0.68, -0.001)** |
| Ward class |  |
| Ward class: A&B1 | -0.54 (-1.13, 0.05) |
| Ward class: B2 | -0.11 (-0.40, 0.19) |
| Ward class: C | - |
| Modified Rankin Scale |  |
| Modified Rankin Scale: No symptom or disability | - |
| Modified Rankin Scale: slight or moderate disability | 0.17 (-0.16, 0.49) |
| Modified Rankin Scale: moderate severe or severe disability | 0.45 (0.06, 0.84)** |
| First-time stroke | 0.03 (-0.32, 0.37) |
| NIHSS: moderate to severe stroke | 0.16 (-0.11, 0.44) |
| Cardiovascular disease | -0.01 (-0.34, 0.32) |
| Diabetes | 0.10 (-0.16, 0.37) |
| Hypertension | 0.20 (-0.11, 0.51) |
| Hyperlipidemia | -0.08 (-0.42, 0.26) |
| Depressed | 0.32 (0.05, 0.59)** |
| Caregivers' Age |  |
| Caregivers' age group: <= 50 | - |
| Caregivers' age group: 51 - 65 | 0.06 (-0.24, 0.36) |
| Caregivers' age group: > 65 | -0.27 (-0.67, 0.12) |
| Caregivers' gender: Female | 0.42 (0.03, 0.80)** |
| Caregivers’ work status |  |
| Full-time workers | 0.30 (0.01, 0.59)** |
| Part-time workers | -0.01 (-0.43, 0.41) |
| Unemployed and retired | - |
| Hours of care provided |  |
| Hours of care provided: <=28 hours/week | -0.11 (-0.46, 0.24) |
| Hours of care provided: 28 - 56 hours/week | 0.12 (-0.18, 0.42) |
| Hours of care provided: > 56 hours/week | - |
| Co-caring Status |  |
| Co-care with others | 0.05 (-0.26, 0.36) |
| Co-care with foreign domestic workers | -0.60 (-1.02, -0.18)*** |

Notes: * Significant at 0.10; ** significant at 0.05; *** significant at 0.01

Caregivers in this table means the corresponding informal caregivers who were assisting informal caregivers at the same time.

This table shows the results considering three categories for the employment status of the assisting informal caregivers: full-time workers, part-time workers, unemployed and retired.

**Figure I**: Hours of Informal Care Required by Patients at Month 3 and Month 12


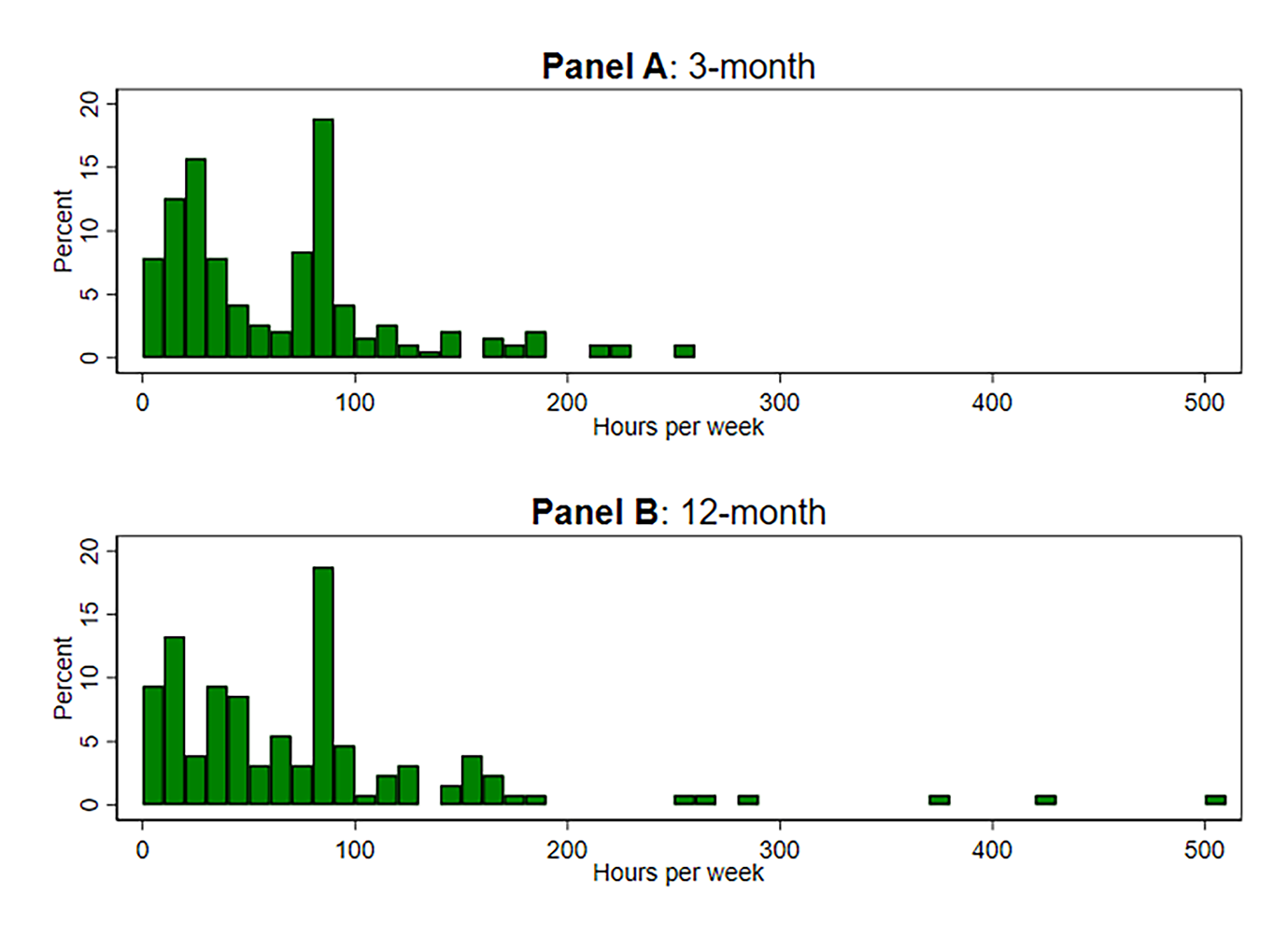


**Figure II**: Zarit’s Burden Score of Informal Caregivers at Month 3 and Month 12


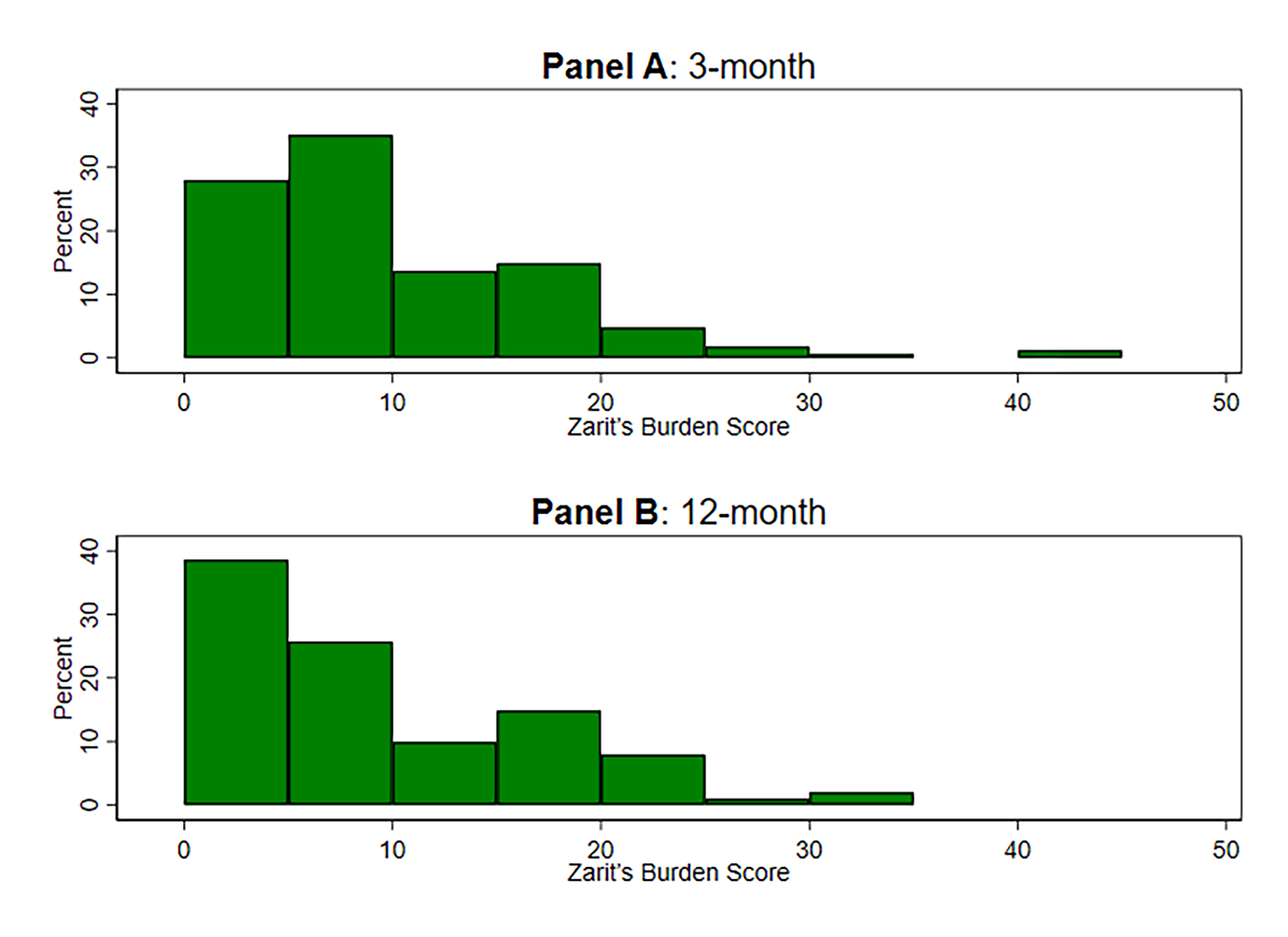


Method: Regression Analysis

For hours of care received by the patients, the analysis was conducted in two steps. First, logistic regression was applied to examine what factors were associated with the requirement of informal care. The value of dependent variable was 1 for the patients that required informal care, and 0 otherwise. Second, generalized linear regression with Gamma distribution and log link was applied using hours of informal care among patients who required informal care. In the full model, the control variables included patients’ demographic information, patients’ MRS, first-time stroke, NIHSS at recruitment, comorbidities at recruitment, and whether patients feeling depressed.

For Zarit’s Burden Score, generalized linear regression with Gamma distribution and log link was used. In the full model, besides the control variables used in the regression analysis of hours of informal care, additional control variables included: caregivers’ demographic information, caregivers’ working status, hours of informal care provided, co-care with others, and co-care with FDWs. Informal caregivers’ ethnicity and marital status were not included as these two variables were highly collinear with the ethnicity and marital status of patients.
